# The novel circular RNA HIPK3 accelerates the proliferation and invasion of hepatocellular carcinoma cells by sponging the micro RNA-124 or micro RNA-506/pyruvate dehydrogenase kinase 2 axis

**DOI:** 10.1080/21655979.2022.2031398

**Published:** 2022-02-25

**Authors:** Qiangfeng Yu, Wenxiang Chen, Yiming Li, Jun He, Yu Wang, Sijia Yang, Jianyin Zhou

**Affiliations:** a The Second Department of General Surgery, Zhuhai People’s Hospital, Zhuhai, China; b Department of Hepatobiliary and Pancreatic Surgery, Zhongshan Hospital, Xiamen University, Xiamen, China

**Keywords:** circHIPK3, hepatocellular carcinoma, miR-124, miR-506, pyruvate dehydrogenase kinase 2

## Abstract

Circular RNAs (circRNAs) have been confirmed to be associated with the progression of various cancers, including hepatocellular carcinoma (HCC). However, the role and mechanism of circHIPK3 in HCC are still unclear. To investigate its function, circHIPK3 expression was first determined by RT–qPCR in HCC tissues or cells. Functionally, cell proliferation and invasion were investigated by CCK-8, EdU, or Transwell assays. In terms of understanding the mechanism, the interaction of the circRNA HIPK3/micro RNA 124 (miRNA 124) or micro RNA 506 (miRNA506) /PDK2 regulatory loop was verified by dual-luciferase reporter gene assay. In addition, a xenograft tumor model was established to confirm the impact of circHIPK3 on the growth of HCC cells *in vivo*. We found that circHIPK3 was upregulated in HCC patients and associated with clinical characteristics, while miR-124 and miR-506 were downregulated in HCC patients. Additionally, we proved that knock down of circHIPK3 remarkably suppressed the proliferation and invasion of HCC cells. Mechanistically, circHIPK3 directly bound to miR-124 or miR-506 and inhibited their expression, and PDK2 was a target gene of miR-124 or miR-506. Moreover, circHIPK3 overexpression reversed the inhibitory effect of miR-124 or miR-506 on HCC progression. miR-124 or miR-506 could also suppress tumorigenesis of HCC cells by PDK2. Furthermore, *in vivo* evidence confirmed that knock down of circHIPK3 inhibited tumor formation. We suggest that circHIPK3 can accelerate the proliferation and invasion of HCC cells by sponging miR-124 or miR-506 to upregulate PDK2, which is the underlying mechanism of circHIPK3-induced HCC progression.

## Introduction

Liver cancer is the third leading cause of cancer-related death worldwide after lung and colorectal cancer [1]. Hepatocellular carcinoma (HCC) accounts for 85% to 90% of primary liver cancer and is a highly invasive malignant tumor [2]. Risk factors for HCC mainly include chronic hepatitis B and C, alcoholic liver disease, metabolic liver disease, exposure to dietary toxins, etc [3,4]. Statistically, approximately 750,000 patients die of HCC each year globally [5]. The incidence of HCC is geographically heterogeneous, with approximately 85% of HCC occurring in developing countries and underdeveloped areas [6]. Currently, liver transplantation and liver resection are two effective treatments for HCC [7]. Due to the high degree of malignancy and poor prognosis of HCC, most patients do not have the opportunity for surgical treatment since most cases are found in an advanced stage [8]. HCC has a high degree of malignancy and poor prognosis. Most patients are first diagnosed at an advanced stage and have lost the opportunity for surgical treatment [9]. Consequently, there is an urgent need to find potential therapeutic targets for HCC therapy.

CircRNAs are a group of RNAs that regulate transcriptional and posttranscriptional gene expression and are produced from the back-splicing of RNA [10,11]. Due to the specificity and stability of circRNAs, they play a key role in the development and metastasis of different cancers. Recently, many circRNAs have been proven to be relevant to the progression of HCC, such as circ_101280 [12], circ_0051443 [13], circ_0000517 [14], circ_0000517 [15], Circ_0001955 [16], and circ_0000285 [17]. CircHIPK3 dysregulation has also been discovered as an oncogenic mechanism in multiple cancers, including lung cancer [18], renal cancer [19], gastric cancer [20], bladder cancer [21], prostate cancer [22], pancreatic cancer [23], and thyroid cancer [24]. However, the function and possible mechanism of circHIPK3 in HCC progression are largely unknown.

It has been reported that circRNAs possess microRNA (miRNA) response elements (MREs) [25]. CircRNAs, as competitive endogenous RNAs (ceRNAs), can competitively bind miRNAs and block their binding to targets, thereby affecting the expression of target genes [26]. CircRNAs can act as sponges to absorb miRNAs and regulate their functions, which is a key mechanism by which circRNAs affect cancer progression [27,28]. Recent investigations have reported that miR-124-3p acts as an important tumor-suppressive miRNA to suppress HCC carcinogenesis through targeting CRKL [29]. To investigate the possible mechanism of circHIPK3 in HCC, we also performed bioinformatics analysis to predict the targeted miRNAs of circHIPK3. In accordance with the predicted results, we unexpectedly discovered that there were latent binding sites between circHIPK3 and miR-124-3p or miR-506-3p. Therefore, we speculated that circHIPK3 might alter HCC progression by targeting miR-124-3p and miR-506-3p.

In this study, we aimed to determine the expression and roles of circHIPK3, miR-124-3p and miR-506-3p in HCC patients and HCC cells. In addition, we explored the possible regulatory mechanism of circHIPK3 on miR-124-3p and the miR-506-3p axis in HCC. Results from this current study reveal the potential value of the circHIPK3/miR-124-3p or miR-506-3p axis in the diagnosis, treatment and prognosis of HCC.

## Materials and methods

### Clinical specimens

Liver cancer and tumor-adjacent tissues were collected from 30 HCC patients who were admitted to Zhongshan Hospital. The clinical information of the 30 patients with HCC was as follows: sex (male 17; female, 13), age(≥55, 16; <55, 14), tumor size (≥4 cm, 14; <4 cm, 16), lymph node metastasis (positive, 13; negative, 17), and TNM stage (I–II, 16; III–IV, 14). Permission was obtained from the ethics committee of Zhongshan Hospital, and all participants signed written informed consent.

### Cell lines

Liver cancer cell lines (HepG2, SMMC-7721, Bel-7402 and Huh-7) and a human hepatic cell line (HL7702) were purchased from ATCC. SMMC-7721, Bel-7402, and HL7702 cells were grown in RPMI 1640 medium (Gibco); HepG2 cells were cultured in MEM (Gibco, Cat. No. 41,500,034); Huh-7 cells were maintained in DMEM (Sigma–Aldrich, Cat. No. D6429). All media were supplemented with 10% fetal bovine serum (Gibco), and all the cells were grown at 37°C and 5% CO_2_.

### Cell transfection

Negative control (NC), circHIPK3 siRNAs (si-circHIPK3), NC mimics, miR-124 mimics, and miR-506 mimics were chemically synthesized by GenePharma (Shanghai, China). Empty vector, circHIPK3 overexpression plasmid and PDK2 overexpression plasmid were obtained from HanBio Biotechnology (Shanghai, China). Based on the experimental purpose, HepG2 and SMMC-7721 cells were transfected with these plasmids or oligonucleotides using Lipofectamine 3000 (Invitrogen) for 48 h by referring to the reagent instructions [30]. This assay was repeated three times.

### Real-time quantitative polymerase chain reaction (RT-qPCR)

Total RNA from HCC tissues and cells was extracted with TRIzol (Invitrogen), and the concentration was calculated by measuring the absorbance at A260/280. After incubation with or without 3 U/μg RNase R, an RNeasy MinElute cleaning kit was used to purify the resulting RNA. RNA (1.5 μg) was reverse transcribed into cDNA with a BestarTM qPCR RT kit (DBI; 2220). PCR amplification was conducted using SYBR Green Master Mix (Roche) on an Applied Biosystems 7500 H system (Foster City, CA, USA) with specific primers. The PCR conditions were as follows: denaturation at 95°C for 20s, 30 cycles of denaturation at 95°C for 30s, annealing at 55°C for 30s and extension at 72°C for 30s [31]. This assay was repeated three times. All primer sequences are displayed in Table 1.

**Table 1.** The sequences of primers in RT-qPCR

ID	Sequence (5’- 3’)
GAPDH	Forward: TGTTCGTCATGGGTGTGAAC
GAPDH	Reverse: ATGGCATGGACTGTGGTCAT
CircHIPK3	Forward: GCTACCACAGGATCAAAACAGA
CircHIPK3	Reverse: CACATAGGTCCGTGGATAGTTT
U6	Forward: CTCGCTTCGGCAGCACA
U6	Reverse: AACGCTTCACGAATTTGCGT
miR-124	Forward: ACACTCCAGCTGGGCGTGTTCACAGCGGAC
	Reverse: CTCAACTGGTGTCGTGGA
miR-506	Forward: ACACTCCAGCTGGGTATTCAGGAAGGTGTT
	Reverse: CTCAACTGGTGTCGTGGA

### Cell counting Kit-8 (CCK-8)

Treated HepG2 or SMMC-7721 cells were seeded in 96-well plates overnight at a concentration of 2 × 10^4^/well. Twenty microliters of CCK-8 solution (Dojindo) were added to each well and incubated at 37°C. The optical density (OD) values were measured at 450 nm every 24 hours by a scanning multiwell spectrophotometer [32]. This assay was repeated three times.

### Cell proliferation assay using 5-ethynyl-2ʹ-deoxyuridine (EdU)

Cell viability was tested with a Cell-Light^TM^ EdU kit (Cat. No. C10311). Transfected HepG2 or SMMC-7721 cells in a 24-well plate were fixed using 4% paraformaldehyde for 20 min, treated with 2 mg/mL glycine for 10 min, and cleared with 0.5% Triton X-100. Then, the cells were processed with 100 μL Apollo solution for 30 min and 100 μL Hoechst 33,342 for 20 min. After washing and permeating, the results were viewed with a fluorescence microscope [33]. This assay was repeated three times.

### Transwell assay

The bottom membrane of the Transwell chamber (Costar; MA, USA) was coated with diluted Matrigel (dilution ratio, 1:8, 50 μL) for 30 min at 37°C. Then, the transfected HepG2 or SMMC-7721 cells were suspended in serum-free medium and inoculated in the upper Transwell chamber, and medium with 20% FBS was added to the lower Transwell chamber. After incubation for 24 h at 37°C, the invaded cells were fixed, stained with 0.1% crystal violet, and photographed with a light microscope (Nikon, Japan) [34]. Five random fields per group were photographed under an optical microscope and the number of cells was counted. This assay was repeated three times.

### Dual luciferase reporter assay

The sites of circHIPK3 and miR-124 or miR-506 were predicted with Starbase (https://starbase.sysu.edu.cn/index.php), and the putative target genes associated with miR-124 or miR-506 were predicted by the TargetScan database (www.targetscan.org/vert_72). Wild-type (WT) or mutant (MUT) circHIPK3 or PDK2 were synthesized and then cloned into pis-Check 2 dual-luciferase reporter vectors (Promega). HepG2 cells were seeded in 24-well plates and cotransfected with miR-124 mimics or miR-506 mimics and WT- or MUT-circHIPK3 or PDK2 using Lipofectamine 2000 (Invitrogen). The relative luciferase activities were determined by the Dual-Luciferase Reporter Assay System (Promega) after 48 h of transfection [35]. This assay was repeated three times.

### Western blot

Proteins extracted from HepG2 or SMMC-7721 cells were separated by SDS–PAGE based on the molecular weight of the proteins. Protein from the gels was transferred to PVDF membranes and incubated with primary antibody (PDK2, abcam, UK) overnight at 4°C after blocking with 5% skim milk. The membranes were subsequently washed with 0.1 M phosphate‐buffered saline (PBS) with Tween‐20 and then incubated with HRP-conjugated secondary antibodies (abcam, UK). Finally, ECL (KeyGen) was adopted to test the immunoreactivities [36]. This assay was repeated three times.

### Tumor xenograft model

SPF experimental animals (BALB/c male nude mice, 4 weeks, 20 ± 2 g) were purchased from Laboratory Animal Center of Sun Yat-sen University. The mice were raised in a specific environment (temperature, 22°C-25°C; humidity, 45%-55%, 12 h light/12 h dark, and freely available food and water). This study was approved by the IACUC, and the experiment was strictly conducted based on IACUC. The transfected HepG2 cell suspension (1 × 10^6^ cells) was inoculated subcutaneously into nude mice. We recorded the longest diameter (L) and shortest diameter (W) of the tumors every 7 days for 28 days. At 28 days, the mice were anesthetized, and the tumors were removed and photographed [37].

## Statistical analysis

The data were analyzed by Student’s t test and One-way analysis of variance using SPSS 22.0 software (SPSS, Chicago, IL, USA). All results are expressed as means ±SD. The statistical significance was set at *P* < 0.05.

## Results

### Silencing of circHIPK3 notably restrained the proliferation and invasion of HCC cells

**Figure 1.**
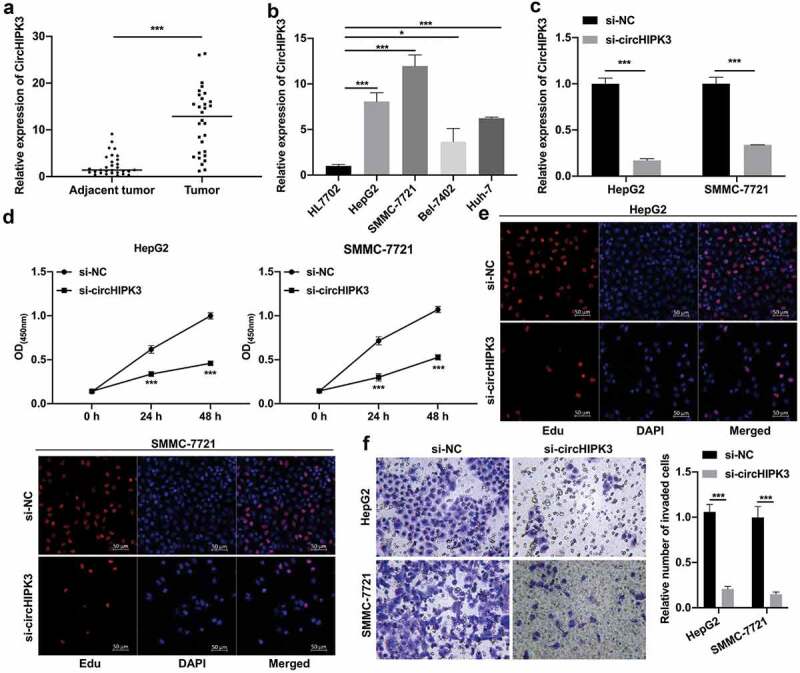
Silencing of circHIPK3 notably restrained the pr**oliferation and invasion of HCC cells**. (a) RT–qPCR assay showed the change in circHIPK3 in HCC and tumor-adjacent tissues (n = 30). (b) RT–qPCR was conducted to confirm circHIPK3 expression in HL7702, HepG2, SMMC-7721, Bel-7402 and Huh-7 cells. (c) The change in circHIPK3 expression was monitored by RT–qPCR in HepG2 and SMMC-7721 cells transfected with si-circHIPK3. (d and e) CCK-8 and EdU assays were performed to assess cell proliferation in circHIPK3-silenced HepG2 and SMMC-7721 cells. (f) Cell invasion was tested by Transwell assay in HepG2 and SMMC-7721 cells after circHIPK3 knockdown, and the number of invaded cells was counted. *P< 0.05, ***P< 0.001.

To investigate the expression and function of circHIPK3 in HCC, we first collected HCC and tumor-adjacent tissues. RT–qPCR data showed that circHIPK3 expression was significantly increased in HCC compared with tumor-adjacent tissues (Figure 1a). In addition, we discovered that circHIPK3 expression was positively related to tumor size, lymph node metastasis and TNM stage, while there was no significant difference in circHIPK3 expression and sex or age in HCC patients (Table 2). Next, our results showed that circHIPK3 was noticeably upregulated in HepG2, SMMC-7721, Bel-7402 and Huh-7 cells relative to that in HL7702 cells, and the level of circHIPK3 was relatively high in HepG2 and SMMC-7721 cells and relatively low in Bel-7402 and Huh-7 cells (Figure 1b). Thus, we selected HepG2 and SMMC-7721 cells with higher circHIPK3 expression for circHIPK3 silencing through transfection with si-circHIPK3. RT–qPCR results showed that circHIPK3 expression was significantly lower in the si-circHIPK3 group than in the si-NC group, indicating the successful knock down of circHIPK3 in HepG2 and SMMC-7721 cells (Figure 1c). Moreover, the results from CCK-8 and EdU assays showed that circHIPK3 knockdown prevented the proliferation of HepG2 and SMMC-7721 cells (Figures 1d and e). Additionally, Transwell data showed that circHIPK3 knockdown caused a remarkable suppression in the invasion capacity of HepG2 and SMMC-7721 cells (Figure 1f). Overall, our data confirmed that knock down of circHIPK3 contributed to the prevention of HCC progression.

**Table 2.** The correlation between circHIPK3 expression and clinicopathologic characteristics in liver cancer

Parameters	No. of cases	CircHIPK3 expression		
		Low (n = 15)	High (n = 15)	*P*-value
**Gender**				*P*= 0.9999
Male	17	9	8	
Female	13	6	7	
**Age**				*P*= 0.9999
≥55	16	8	8	
<55	14	7	7	
**Tumor size (cm)**				***P*= 0.0007**
≥4	14	2	12	
<4	16	13	3	
**Lymph node metastasis**				***P*= 0.0253**
Positive	13	3	10	
negative	17	12	5	
**TNM stage**				***P*= 0.0025**
I–II	16	14	2	
III–IV	14	1	13

### CircHIPK3 overexpression prominently reversed the inhibitory effects of miR-124 on the proliferation and invasion of HCC cells

**Figure 2.**
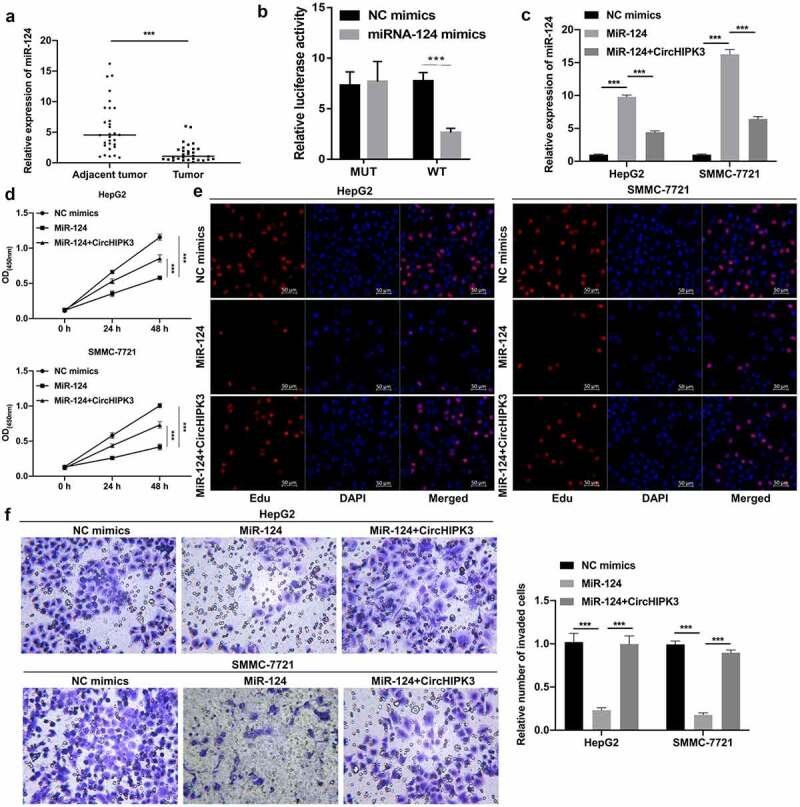
**CircHIPK3 overexpression prominently reversed the inhibitory effects of miR-124 on the proliferation and invasion of HCC cells**. (a) RT–qPCR analysis of miR-124 expression in HCC and tumor-adjacent tissues (n = 30). (b) The interaction between circHIPK3 and miR-124 was analyzed via dual luciferase reporter gene assay in HCC cells. (c) The change in miR-124 expression was confirmed by RT–qPCR in HepG2 and SMMC-7721 cells cotransfected with miR-124 mimics and circHIPK3 plasmids. (d and e) The impact of miR-124 and circHIPK3 on cell proliferation was tested through CCK-8 and EdU assays in HepG2 and SMMC-7721 cells. (f) After cotransfection with miR-124 mimics and circHIPK3 plasmids, a Transwell assay was utilized to determine the invasion capacity of HepG2 and SMMC-7721 cells, and the number of invaded cells was calculated in each region. ***P< 0.001.

Through bioinformatics prediction, we unexpectedly discovered a miRNA (miR-124) that might interact with circHIPK3. Moreover, our results also showed that miR-124 expression was markedly decreased in HCC versus tumor-adjacent tissues (Figure 2a). We further determined the binding ability between circHIPK3 and miR-124, and the data showed that miR-124 mimics could notably decrease the luciferase activity of WT-circHIPK3 but had no effect on the luciferase activity of MUT-circHIPK3 (Figure 2b). Moreover, RT–qPCR data revealed that miR-124 expression was significantly increased in miR-124 mimic-transfected HepG2 and SMMC-7721 cells, while this increased expression of miR-124 was markedly weakened by circHIPK3 overexpression (Figure 2c). Next, CCK-8 and EdU data revealed that miR-124 overexpression significantly diminished cell proliferation, which could also be partially reversed by circHIPK3 overexpression in HepG2 and SMMC-7721 cells (Figure 2d and e). Transwell results revealed that miR-124 overexpression also prominently repressed the invasion of HepG2 and SMMC-7721 cells, which were also dramatically attenuated by circHIPK3 overexpression (Figure 2f). In summary, we discovered that circHIPK3, as a sponge for miR-124, could induce the proliferation and invasion of HCC cells by targeting miR-124.

### CircHIPK3 overexpression markedly attenuated the inhibitory action of miR-506 on the proliferation and invasion of HCC cells

**Figure 3.**
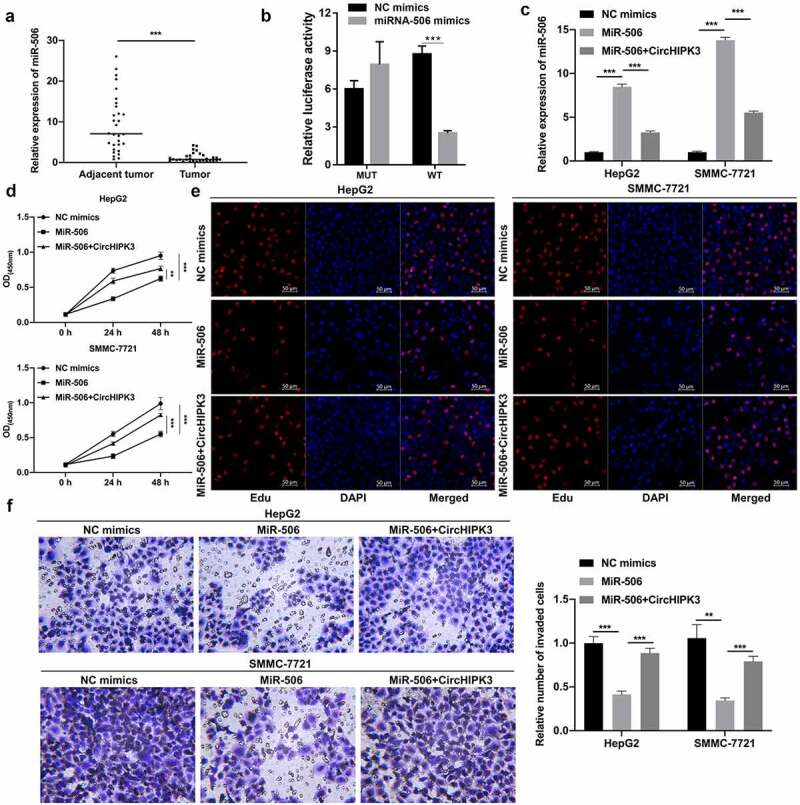
**CircHIPK3 overexpression markedly attenuated the inhibitory action of miR-506 on the proliferation and invasion of HCC cells**. (a) RT–qPCR analysis revealed a change in miR-506 expression in HCC and tumor-adjacent tissues (n = 30). (b) The interaction between circHIPK3 and miR-506 was evaluated using a dual luciferase reporter gene assay. (c) RT–qPCR was performed to confirm the expression change of miR-506 in miR-506- and circHIPK3-overexpressing HepG2 and SMMC-7721 cells. (d and e) CCK-8 and EdU assays revealed a change in cell proliferation of HepG2 and SMMC-7721 cells cotransfected with miR-506 mimics and/or circHIPK3 plasmids. (f) Transwell assays were performed to monitor the influences of circHIPK3 and miR-506 on the invasion ability of HepG2 and SMMC-7721 cells, and the invaded cells were also counted. **P< 0.01, ***P< 0.001.

Furthermore, we also discovered that miR-506 might be another underlying target of circHIPK3. The RT–qPCR results also indicated that the level of miR-506 was notably reduced in HCC tissues compared to tumor-adjacent tissues (Figure 3a). Moreover, our results showed that the luciferase activity of WT-circHIPK3 was noticeably reduced by miR-506 mimics, while the luciferase activity of MUT-circHIPK3 was unaffected by miR-506 mimics; thus, these results indicated that circHIPK3 could target miR-506 (Figure 3b). The RT–qPCR results showed that miR-506 expression was markedly heightened in the miR-506 group compared with the NC group, while this elevation of miR-506 expression was dramatically weakened by circHIPK3 overexpression (Figure 3c). Functionally, we first demonstrated that overexpression of miR-506 notably suppressed cell proliferation, while this suppression mediated by miR-506 overexpression could also be prominently reduced by circHIPK3 overexpression in HepG2 and SMMC-7721 cells (Figure 3d and e). Transwell data also confirmed that miR-506 overexpression could dramatically prevent cell invasion, which was also markedly reversed by circHIPK3 overexpression in HepG2 and SMMC-7721 cells (Figure 3f). Generally, the data proved that circHIPK3 could also promotes HCC progression by targeting miRNA-506.

### Overexpression of PDK2 markedly enhanced tumorigenesis of HCC cells mediated by miR-124 or miR-506

**Figure 4.**
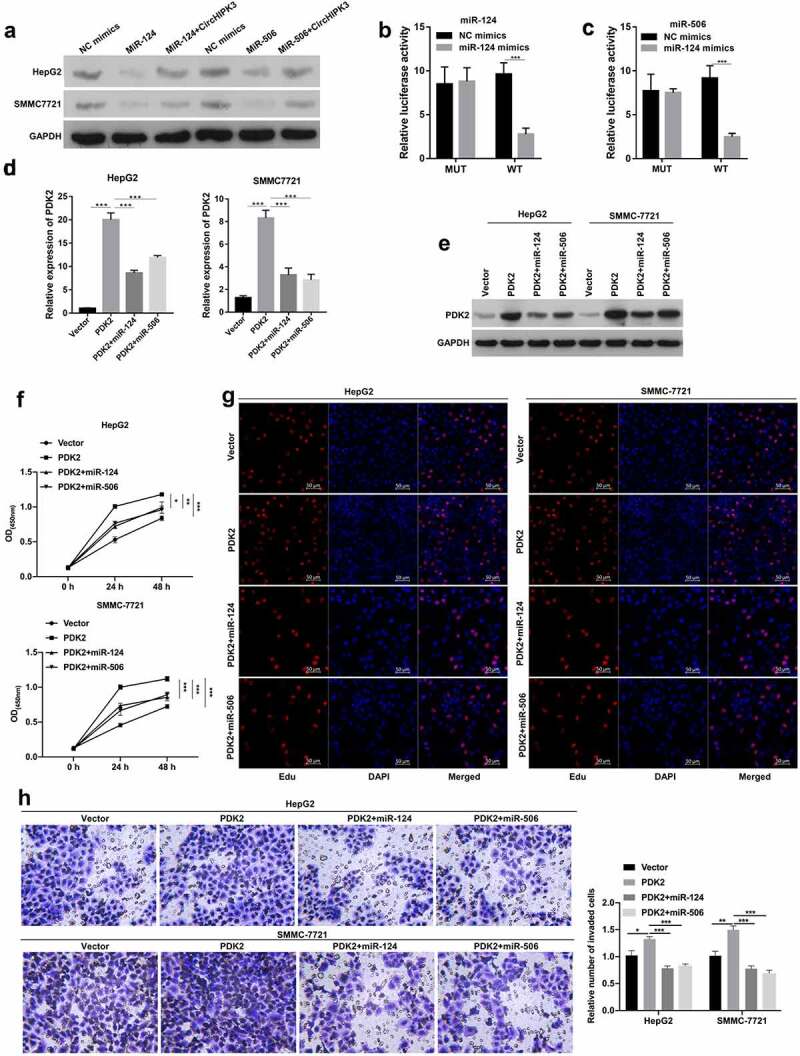
**Overexpression of PDK2 markedly enhanced tumorigenesis of HCC cells mediated by miR-124 or miR-506**. (a) Western blotting analysis revealed the change in expression of PDK2 in HepG2 cells after transfection with miR-124 or miR-506 and circHIPK3 plasmids. (b and c) The interaction between PDK2 and miR-124 or miR-506 was also determined with a dual luciferase reporter assay in HepG2 cells. (d and e) RT–qPCR was conducted to examine PDK2 expression in HCC cells after transfection with the PDK2 plasmid and miR-124 or miR-506. The effects of PDK2 and miR-124 or miR-506 on cell proliferation and invasion were tested with CCK-8 (f), EdU (g) and Transwell assays (h). *P< 0.05, **P< 0.01, ***P< 0.001.

Subsequently, we further predicted the latent target gene of miR-124 or miR-506. First, Western blotting data showed that PDK2 was significantly downregulated in miR-124- or miR-506-transfected HepG2 and SMMC-7721 cells, while the miR-124- or miR-506 overexpression-mediated decrease in PDK2 expression was observably reversed by circHIPK3 overexpression (Figure 4a). Second, the data proved that miR-124 or miR-506 overexpression could markedly reduce the luciferase activity of WT-PDK2 but had no effect on the luciferase activity of MUT-PDK2 (Figure 4b and c). Third, we cotransfected the PDK2 plasmid and miR-124 or miR-506 into HepG2 and SMMC-7721 cells, and our data verified that PDK2 overexpression could notably upregulate PDK2 in HepG2 and SMMC-7721 cells, which was dramatically reduced by miR-124 or miR-506, especially miR-124 (Figure 4d and e). In addition, our results indicated that miR-124 or miR-506 overexpression prominently reversed the inhibitory action of PDK2 overexpression on the proliferation of HepG2 and SMMC-7721 cells (Figure 4f and g). Additionally, miR-124 or miR-506 overexpression markedly attenuated the promoter role by PDK2 on the invasion of HepG2 and SMMC-7721 cells (Figure 4h). Thus, these results revealed that miR-124 or miR-506 could block the malignant biological properties of HCC cells through PDK2.

### Knock down of circHIPK3 dramatically suppressed growth and upregulated miR-124 and miR-506 in HCC xenograft tumors

**Figure 5.**
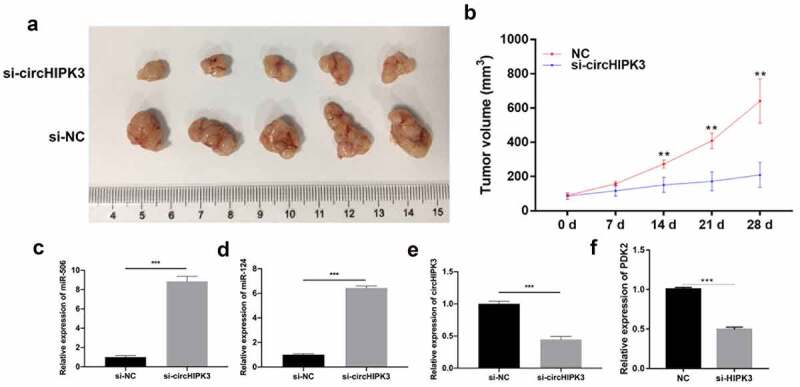
**Knock down** of circHIPK3 **dramatically suppressed growth and upregulated miR-124 and miR-506 in HCC xenograft tumors**. (a) After 4 weeks, groups of tumors were present in nude mice as observed by photography and comparison. (b) The length and width of the tumors were measured, and their volume was calculated at 0, 7, 14, 21 and 28 days. RT–qPCR assays revealed the expression changes of miR-506 (c), miR-124 (d), circHIPK3 (e) and PDK2 (f) in xenograft tumors. *P< 0.05, **P< 0.01, ***P< 0.001.

Based on the role and mechanism of circHIPK3 in HCC cells, we further verified the effect of circHIPK3 in HCC xenograft tumors. As shown in Figure 5a, tumors in nude mice were observably diminished in the si-circHIPK3 group compared with the si-NC group. Silencing circHIPK3 also significantly reduced the volume of xenograft tumors (Figure 5b). Additionally, RT–qPCR results revealed that knock down of circHIPK3 could prominently upregulate miR-124 and miR-506 and downregulate PDK2 and circHIPK3 (Figures 5c-f). Overall, these data revealed that circHIPK3 knockdown could also prevent the growth of HCC in nude mice and regulate miR-124, miR-506, and PDK2.

## Discussion

HCC is one of the most universal malignant tumors of the digestive system worldwide and has not been fully and effectively controlled [38]. Currently, finding new molecular targets to explore more effective therapeutic strategies has become an urgent challenge for HCC therapy. CircRNAs, as endogenous noncoding RNAs, have a covalently circular closed single-stranded RNA structure [39]. Recently, researchers have gradually recognized the crucial role of circRNAs in the life sciences [40]. CircHIPK3 (hsa_circ_0000284) is located on chr11:33,307,958–33,309,057 and is derived from the HIPK3 gene [41]. In the literature, circHIPK3 has been shown to contribute to the acceleration of the progression of multiple cancers. For instance, loss of circHIPK3 could accelerate autophagy of lung cancer cells [18]. Furthermore, circHIPK3 could induce tumorigenesis of renal cancer cells [19], enhance invasion of thyroid cancer [24], and also facilitate growth, metastasis and oxaliplatin-resistance of colorectal cancer cells [42,43]. The biological function of circHIPK3 in HCC has been reported in present studies, suggesting that circHIPK3 may contribute to HCC [44–46]. In our study, we demonstrated that circHIPK3 was significantly upregulated in HCC tissues. Knock down of circHIPK3 also notably prevented the proliferation and invasion of HCC cells. Thus, we speculated that circHIPK3 could also accelerate the tumorigenesis of HCC cells.

CircRNAs act a miRNA sponges and play a crucial role in translation regulation [10,47]. A close association between circRNAs and miRNAs has been confirmed in various cancers [48]. miRNAs are a type of endogenous, short and highly conserved small noncoding RNA [49,50]. miRNAs can regulate gene expression at the posttranscriptional stage by binding to the 3’-UTR of mRNAs [51]. Many studies have shown that abnormal expression of miRNAs is associated with cancer processes, including proliferation, metastasis and apoptosis [52,53]. Through bioinformatics analysis, we unexpectedly discovered that there were potential binding sites between circHIPK3 and miR-124 or miR-506, indicating that miR-124 or miR-506 might be targeted by circHIPK3. miR-124 has also been reported to inhibit several cancer processes, including breast cancer [54], gastric cancer [55], and bladder cancer [56]. Furthermore, miR-506 has also been proven to act as a tumor suppressor in multiple cancers, such as thyroid cancer [57], cervical cancer [58],colorectal cancer [59] and HCC [60]. Moreover, we discovered that miR-124 could delay hepatic carcinogenesis by interacting with miR-506 [61]. Previous study revealed that circHIPK3 regulates AQP3 expression via miR-124 [46]. However, it is not known whether circHIPK3 can accelerate the process of HCC through miR-124 and miR-506. In our study, we further confirmed that circHIPK3 can act as a sponge by regulating the expression of miR-124 and miR-506. In addition, circHIPK3 could also induce the proliferation and invasion of HCC cells by targeting miR-124 and miR-506. Therefore, we proved that in the progression of liver cancer formation, miR-124 and miR-506 could act as tumor suppressors, and miR-124 and miR-506 were the downstream regulatory targets of circHIPK3 in HCC.

Research has also demonstrated that circRNAs can prevent the inhibitory effect of miRNAs on mRNAs by sponging miRNAs [62]. Although the basic biological roles of the circHIPK3/miR-124 or miR-506 axis in HCC were preliminarily investigated in this study, the mechanism of miR-124 and miR-506 was not further studied. Through bioinformatics prediction, we further discovered that PDK2 might be a possible target gene of miR-124 and miR-506. Thus, we speculated that circHIPK3 could also regulate the target gene (PDK2) of miR-124 and miR-506 by acting as a competitive endogenous RNA (ceRNA). Related studies have verified that tumor cells also rely on the glycolysis pathway as their main energy source even under normal oxygen content [63,64]. Pyruvate dehydrogenase kinases (PDKs), as key regulators of the aerobic glycolysis pathway, can change cell metabolism by affecting aerobic glycolysis and participate in malignant cell transformation [65]. Among them, PDK2 was most closely associated with tumors. PDK2 is abnormally overexpressed in multiple tumors, which is relevant to the malignant phenotype of tumors [66,67]. In addition, research confirmed that PDK2 is upregulated in HCC, and downregulation of PDK2 can suppress the proliferation and metastasis of HCC cells [68].

In our current study, we further confirmed that as the target gene of miR-124 or miR-506, PDK2 can also be involved in the prevention of miR-124- or miR-506-mediated liver cancer cell proliferation and invasion. Therefore, we further proved the role of the miR-124 or miR-506/PDK2 axis in HCC cells.

## Conclusions

Our study indicated a novel regulatory loop involving the circHIPK3/miR-124 or miR-506/PDK2 axis in HCC progression. These data suggested that circHIPK3 might be a novel therapeutic target for HCC therapy.

## Data Availability

The data and material are available from the corresponding author upon reasonable request.

## Article highlights

circHIPK3 inhibited proliferation and invasion of HCC

miRNA-124/506 directly target PDK2

circHIPK3 positively regulates PDK2 expression by sponging miRNA-124 and miRNA-506
